# Impact of COVID-19 pandemic on ophthalmic presentations to an Australian outer metropolitan and rural emergency department: a retrospective comparative study

**DOI:** 10.1186/s12886-022-02271-8

**Published:** 2022-01-28

**Authors:** King Fai Calvin Leung, Mojtaba Golzan, Chaminda Egodage, Simon Rodda, Richard Cracknell, Peter Macken, Shweta Kaushik

**Affiliations:** 1 Department of Emergency Medicine, Campbelltown Hospital, Therry Road, Campbelltown, New South Wales 2560 Australia; 2 Vision Science group, Graduate School of Health (GSH), University of Technology Sydney (UTS), Chippendale, New South Wales Australia; 3 Department of Emergency Medicine, Bowral and District Hospital, Bowral, New South Wales Australia; 4 Department of Ophthalmology, Bowral and District Hospital, Bowral, New South Wales Australia; 5 Department of Ophthalmology, Campbelltown Hospital, Campbelltown, New South Wales Australia

**Keywords:** COVID-19, Epidemiology, Emergency medicine, Ophthalmic emergencies, Tele-ophthalmology

## Abstract

**Background:**

To analyse ophthalmic presentations to an outer metropolitan and a rural emergency department (ED) during the first wave of the COVID-19 pandemic in New South Wales (NSW), Australia.

**Methods:**

A retrospective comparative study of ophthalmic emergency presentations to Campbelltown Hospital (fifth busiest NSW metropolitan ED; population 310,000) and Bowral and District Hospital (rural ED; population 48,000) before and during COVID-19 was conducted. Patient demographics, triage category, referral source, diagnosis, length of stay, departure status, and follow-up location were assessed from coding data between March 1st to May 31st in 2019 and 2020, corresponding to the peak case numbers and restrictions during the first wave of the COVID-19 pandemic in NSW. Differences before and during COVID-19 were analysed using chi-squared tests or independent sample t-tests.

**Results:**

There was no change in ophthalmic presentations at Campbelltown (*n* = 228 in 2019 vs. *n* = 232 in 2020; + 1.75%, *p* = 0.12) and an increase at Bowral (*n* = 100 in 2019 vs. *n* = 111 in 2020; + 11%, *p* < 0.01) during COVID-19. Urgent ophthalmic presentations (Triage Category 3) decreased at Bowral (*p* = 0.0075), while non-urgent ophthalmic presentations (Triage Category 5) increased at both hospitals (Campbelltown *p* < 0.05, Bowral *p* < 0.01).

**Conclusions:**

There was no change in the total number of ophthalmic presentations to an outer metropolitan and an increase to a rural ED during the first wave of the COVID-19 pandemic in New South Wales, Australia. A change in the type of ophthalmic presentations at these peripheral EDs suggest that a high demand for ophthalmic services remained despite the pandemic and its associated gathering and movement restrictions. A flexible healthcare delivery strategy, such as tele-ophthalmology, may optimise patient care during and after COVID-19.

**Supplementary Information:**

The online version contains supplementary material available at 10.1186/s12886-022-02271-8.

## Background

The COVID-19 pandemic has significantly impacted health care systems worldwide and resulted in radical changes to service provision. Since the introduction of lockdowns and other restrictions at the height of the pandemic, there has been a general reduction in adult and paediatric attendances to emergency departments (EDs) [1–11]. The fear of contagion and social gathering restrictions in response to SARS-CoV-2 may have caused patients to avoid seeking medical attention or may have limited their access to care for non-COVID-19 illnesses [7, 12]. A similar trend has been observed for eye-care with various studies reporting a reduction in ophthalmic-related presentations to EDs [13–20].

SARS-CoV-2 was first identified in Wuhan, China, in December 2019 [21]. By 30th January 2020, this novel virus had spread across 19 countries, with the World Health Organisation declaring the COVID-19 outbreak as a pandemic on 11th March 2020 [21]. The first case was recorded in Melbourne, Australia on 25th January 2020, with three other cases identified on the same day in Sydney, New South Wales (NSW) [22, 23]. Subsequently, there was a rise in COVID-19 cases in the first wave of the pandemic in NSW during March.

However, in comparison to other countries, the closure of international borders in Australia on 20th March, as well as the introduction of public event and gathering restrictions from the 16th March and the Public Health Order for Restrictions on Gathering and Movement by the NSW Government on 30th March [24], lead to a plateauing of new COVID-19 diagnoses during the peak of the first wave between late March to May in NSW [25]. These stay-at-home restrictions instructed NSW citizens to not leave their place of residence without a reasonable excuse and not participate in gatherings in a public place of more than 2 persons [24]. Multiple public premises, including restaurants, pubs, indoor recreation facilities, and entertainment facilities were closed. Reasonable excuses for leaving a place of residence included obtaining food or other goods and services, travelling for work or education if unable to do so at home, exercise, and seeking medical or compassionate care. Essential services and occupations such as emergency services, transportation, and supermarkets remained in operation, and thus workers in these fields were deemed essential and allowed to leave home for work. Easing of restrictions started on the 15th May, with re-opening of most premises from the 1st June.

The ocular surface has been proposed as a possible site of exposure and infection by SARS-CoV-2 [26–28], with COVID-19 patients developing ocular surface symptoms and follicular conjunctivitis [28, 29]. The Ophthalmological community responded in part by reinforcement of personal protective equipment (PPE) use (including the use of slit lamp breath shields), change in triaging for operative procedures, and the increased use of telemedicine services [30–32] to reduce transmission. In our emergency departments, increased sanitisation of patient assessment areas and ophthalmic equipment; rapid treatment and discharge of low acuity patients in the triage areas; isolating patients with respiratory symptoms; use of protective eyewear and face shields, N95 face masks and gloves for clinical staff; as well as surgical masks for patients; were precautions used to reduce transmission of SARS-CoV-2, particularly via the respiratory and proposed ocular routes of transmission [33]. Elective operative procedures were cancelled across Australia during late March 2020 [34]. Pre-COVID-19 studies have found that patients commonly present to the ED with conditions that may be managed in a primary care setting, such as conjunctivitis, corneal abrasion, keratitis, iridocyclitis, or symptoms secondary to cataracts [35, 36]. However, a reduced access to primary care providers was observed in NSW during the first wave of the pandemic [11]. Whilst access to emergency departments was largely unaffected by the stay-at-home orders in NSW, it has been proposed that the anxiety of presenting to the ED during this period may have led to a reduction in ophthalmic presentation numbers at several metropolitan Australian EDs [15, 37].

It is unclear if this pattern of presentations is replicated in other busier, outer metropolitan locations or in rural EDs across NSW. With several reports suggesting a growing trend of metropolitan workers relocating to regional areas in NSW during the pandemic, it would be valuable to measure whether such redistribution of population had an impact on local EDs. An understanding of how the COVID-19 pandemic and its associated restrictions in NSW had affected the presentation and management of patients with ophthalmic conditions to both metropolitan and rural EDs in Australia may assist in appropriate medical resource allocation during pandemics and reveal areas that may be enriched by new and innovative approaches to ophthalmic care delivery. The aim of this study was to assess the changes in ophthalmic presentations to an outer metropolitan ED and a rural ED during the period corresponding to the peak case numbers and restrictions for the first wave of the COVID-19 pandemic in NSW, from March 1st to May 31st in 2019 and 2020.

## Methods

Our study focused on two EDs, Campbelltown Hospital and Bowral and District Hospital, located within South Western Sydney Local Health District (SWSLHD). SWSLHD covers seven Local Government Areas (LGA), serving a population of approximately 820,000 people. Campbelltown Hospital ED (Modified Monash 1) is the major 40-bed metropolitan Level 5 emergency service for the Macarthur area, which includes the LGAs of Campbelltown, Camden, and Wollondilly Shire, with a population close to 310,000. Bowral and District Hospital ED (Modified Monash 3) is a Level 3 rural ED serving the Southern Highlands region with a population of around 48,000. Its ED has 10 beds. The Modified Monash Model is used by the Australian Government to define whether a location is metropolitan (Modified Monash 1) or very remote (Modified Monash 7).

Patients presenting with an ophthalmic-related issue to either EDs are first reviewed by the emergency doctor and referred to the on-call Ophthalmologist if required. For Campbelltown Hospital, this occurs during work hours (for review in private rooms or in hospital), or after-hours to the ophthalmology registrar at the linked Tertiary referral facility in the Local Health District (Liverpool Hospital), which has an outpatient eye clinic. During workhours and after-hours, ophthalmic referrals from Bowral ED are sent to a single ophthalmology practice for review in the private rooms or in hospital.

A list of ophthalmic-related presentations from these two SWSLHD EDs were retrospectively obtained from the electronic medical records and ED coding data. Dates of inclusion were from 1st March to 31st May for 2019 and 2020. Further visit data were obtained from auditing of individual patient notes. Variables obtained included patient demographics, triage category for each presentation, discharge diagnosis (based on International Classification of Diseases, tenth revision [ICD-10] and SNOMED CT terminology), length of stay in the emergency department, source of referral, departure status, follow-up disposition at discharge, and whether the patient was referred to an Ophthalmology service. Similar discharge diagnoses were grouped together. This includes all ocular surface foreign bodies as “foreign body”, changes in vision as “visual disturbance”, and bacterial or viral conjunctivitis as “acute conjunctivitis”. To avoid an exhaustive list of diagnoses, only diagnoses that had 3 or more presentations in 2019 or 2020 were included.

Data analysis was performed in Microsoft Excel v16.0. Descriptive statistics including percentage change in presentations between 2019 and 2020 were calculated. Percentage change was calculated by dividing the difference between the numbers in 2019 and 2020 with the number in 2019 for each variable. Comparison groups between 2019 and 2020 were analysed with chi-squared tests or independent samples t-tests.

This study was approved by the South Western Sydney Local Health District Human Research Ethics Committee (2020/ETH02045) and was conducted in accordance with the tenets of the Declaration of Helsinki. All patient details were anonymised during data collection. As a retrospective study, a consent waiver was granted by the South Western Sydney Local Health District Human Research Ethics Committee.

## Results

A significant decrease in the total number of presentations to both EDs in 2020 (compared with 2019) was observed (21,314 in 2019 vs. 18,054 in 2020 at Campbelltown ED [15.3% decrease], and 5123 in 2019 vs. 4178 at Bowral ED [18.45% decrease]; both *p* < 0.01; Table 1). However, there was no reduction in the number of ophthalmic-related emergency presentations at Campbelltown Hospital ED during the height of the COVID-19 pandemic (228 in 2019 vs. 232 in 2020, 1.75% increase, *p* = 0.12, Table 1). On the other hand, there was an 11% increase in ophthalmic presentations at Bowral Hospital ED from March to May in 2020 (100 in 2019 vs. 111 in 2020, *p* < 0.01). Both hospitals had an increase in non-urgent (Triage Category 5) ophthalmic presentations (*p* = 0.03 for Campbelltown, *p* < 0.01 for Bowral), whilst Bowral hospital also had a 50% decrease in urgent (Triage Category 3) ophthalmic-related presentations in March to May of 2020 (*p* = 0.0075). There was no change in presentation numbers for other triage categories at both EDs (all *p* > 0.05, Table 1).

**Table 1:** Ophthalmic-related presentation characteristics at Campbelltown and Bowral Emergency Departments (March-1st to May-31st, 2019 and 2020)

	Campbelltown Hospital				Bowral and District Hospital			
	2019	2020	% change	***p*** value	2019	2020	% change	***p*** value
Ophthalmic-Related Presentations (n)	228	232	1.75	0.12	100	111	11	0.00059*
Total Department Presentations (n)	21,314	18,054	−15.30	< 0.01*	5123	4178	−18.45	< 0.01*
Age (mean ± SD; years)	32 ± 22	35 ± 21	9.07	0.075	38 ± 20	42 ± 23	12.10	0.061
Gender (females; n)	86	77	−10.47	0.41	33	35	6.06	0.85
Aboriginal and Torres Strait Islander (n)	11	11	0	0.97	4	6	50	0.64
Triage Category (n)								
*1 – Resuscitation*	0	0	0	–	0	0	0	–
*2 – Emergency*	7	7	0	0.97	2	3	50	0.74
*3 – Urgent*	54	51	−5.56	0.70	32	16	−50	0.0075*
*4 – Semi-Urgent*	128	113	−11.72	0.27	63	73	15.87	0.80
*5 – Non-urgent*	39	61	56.41	0.03*	3	19	533.33	0.0015*
Length of Stay (mean ± SD; minutes)	229 ± 162	148 ± 95	−35.52	< 0.01*	128 ± 91	119 ± 92	−7.082	0.24
Source of Referral (n)								
*GP/Dentist*	59	63	6.78	0.79	7	2	− 71.43	0.068
*Self/Family/Friends*	160	153	−4.38	0.58	90	107	18.89	0.63
*Employer*	1	3	200	0.33	–	–	–	–
*Nursing Home*	1	0	−100	0.31	–	–	–	–
*Community Health*	1	0	−100	0.31	–	–	–	–
*Emergency Dept*	0	3	–	0.09	2	0	−100	0.14
*Other Hospital*	2	3	50	0.67	0	1	–	0.34
*Outpatient Department*	1	0	−100	0.31	–	–	–	–
*Specialist*	0	3	–	0.09	–	–	–	–
*Other*	3	4	33.33	0.72	1	1	0	0.94

Legend: * = statistically significant

At Campbelltown Hospital ED, the number of patients who did not wait to be seen or left at their own risk with ophthalmic presentations decreased during COVID-19 (*p* = 0.049 and 0.0091, Table 2), and the average length of stay also reduced in 2020 for those with ophthalmic conditions (*p* < 0.01, Table 1). There was no change in numbers for other departure statuses at Campbelltown ED (all *p* > 0.05, Table 2). Similarly, there was no significant change in all departure statuses and length of stay for ophthalmic presentations at Bowral and District Hospital ED (all *p* > 0.05, Tables 1 and 2). Average length of stay decreased at Bowral and District Hospital ED in 2020, but this did not reach significance (*p* = 0.24). Source of referral of patients at both EDs did not change (all *p* > 0.05, Table 1).

**Table 2:** Ophthalmic-related presentation disposition patterns at Campbelltown and Bowral Emergency Departments (March-1st to May-31st, 2019 and 2020)

	Campbelltown Hospital				Bowral and District Hospital			
	2019	2020	% change	***p*** value	2019	2020	% change	***p*** value
Departure status (n)								
*Treatment complete*	162	188	16.05	0.22	88	98	11.36	0.98
*Admitted to ED Short Stay Unit*	0	2	–	0.16	**–**	**–**	**–**	**–**
*Admitted to ward*	20	18	−10	0.71	1	1	0	0.94
*Transferred to another hospital*	2	5	150	0.27	0	2	–	0.18
*Did not wait to be attended*	10	3	−70	0.049*	11	10	−9.091	0.65
*Left at own risk*	34	16	−52.94	0.0091*	**–**	**–**	**–**	**–**
Follow-up Location (n)								
*GP/LMO*	106	121	14.15	0.39	56	49	−12.5	0.22
*AMO/Specialist*	19	12	−36.84	0.19	17	21	23.53	0.74
*Medical Practice (not Psych)*	14	10	−28.57	0.39	0	1	–	0.34
*Outpatients (non-psychiatric)*	28	37	32.14	0.30	1	1	0	0.94
*Review in ED - Planned*	5	2	−60	0.25	3	2	−33.33	0.57
*Review in ED - PRN*	9	14	55.56	0.32	8	12	50	0.51
*Not referred*	31	15	−51.61	0.016*	13	22	69.23	0.22
*Unknown*	7	2	−71.43	0.090	**–**	**–**	**–**	**–**
*Other*	9	19	111.11	0.065	2	3	50	0.74
Referred to Ophthalmology Service (n)	58	63	8.62	0.72	18	16	−11.11	0.52

Legend: * = statistically significant

Patients who were not referred to any follow-up service at discharge decreased at Campbelltown Hospital ED during COVID-19 (*p* = 0.016, Table 2). However, this result may be explained by the fact that there was also a reduction in patients who had left at their own risk or did not wait to be seen (who were not referred for follow-up as they left prior to ED medical assessment). There was no significant change in all other follow-up patterns including referral to an ophthalmology service at both hospitals during COVID-19 (all *p* > 0.05, Table 2). There was no change in patient demographics (age, gender, proportion identifying as Aboriginal and Torres Strait Islanders) between the two time points at both Campbelltown and Bowral EDs (all *p* > 0.05, Table 1).

At both hospitals and during both 2019 and 2020, the main discharge diagnoses included anterior segment issues such as foreign bodies, corneal abrasions, conjunctivitis, corneal chemical injuries, eye pain, and periorbital cellulitis (Table 3). There was a significant decrease in the number of patients presenting with visual disturbance at Campbelltown Hospital ED in 2020 (14 vs. 4, − 71% change, *p* = 0.017, Table 3). There were also more periocular allergic reactions at Campbelltown Hospital ED in 2020 (*p* = 0.047). There were no significant changes in the discharge diagnoses of ophthalmic presentations to Bowral and District Hospital ED.

**Table 3:** Ophthalmic-related discharge diagnoses at Campbelltown and Bowral Emergency Departments (March-1st to May-31st, 2019 and 2020)

Campbelltown Hospital					Bowral and District Hospital				
Discharge Diagnosis	2019	2020	% change	***p*** value	Discharge Diagnosis	2019	2020	% change	***p*** value
*Foreign body*	39	58	48.72	0.065	*Foreign body*	24	29	20.83	0.76
*Corneal abrasion*	18	20	11.11	0.787	*Corneal abrasion*	10	14	40.00	0.57
*Acute conjunctivitis*	13	19	46.15	0.312	*Chemical injury to cornea*	3	8	166.67	0.18
*Periorbital cellulitis*	9	15	66.67	0.237	*Eye pain*	5	4	−20.00	0.62
*Eye pain*	12	12	0.00	0.966	*Red eye*	3	4	33.33	0.81
*Chemical injury to cornea*	9	12	33.33	0.539	*Acute conjunctivitis*	6	3	−50.00	0.25
*Red eye*	4	8	100.00	0.261	*Eye swelling*	0	3	–	0.10
*Corneal ulcer*	3	5	66.67	0.495	*Headache*	2	3	50.00	0.74
*Visual disturbance*	14	4	−71.43	0.017*	*Periorbital cellulitis*	4	3	−25.00	0.61
*Laceration of eyelid*	9	4	− 55.56	0.156	*Subconjunctival haemorrhage*	2	3	50	0.74
*Blunt injury of eye*	8	4	−50.00	0.236	*Blunt injury of eye*	3	2	−33.33	0.57
*Welder’s flash*	1	4	300.00	0.186	*Visual disturbance*	3	1	−66.67	0.27
*Allergic reaction*	0	4	–	0.047*					
*Allergic conjunctivitis*	2	3	50.00	0.669					
*Burn of eye*	1	3	200.00	0.326					
*Stye*	7	2	−71.43	0.090					
*Discharge from eye*	5	2	−60.00	0.247					
*Eye swelling*	4	1	−75.00	0.173					
*Blepharitis*	3	0	−100.00	0.081					

Legend: * = statistically significant

## Discussion

In this retrospective study, we investigated the impact of the COVID-19 pandemic on ophthalmic presentations to the ED of a busy outer metropolitan and a rural hospital during the peak of the first wave of infections in NSW. During this period, stay-at-home and restricted public gathering orders were in place across NSW, which did not differ at the Bowral and Campbelltown regions [24]. This public health order instructed individuals to not leave their place of residence except for reasonable excuses such as seeking medical care. Access to hospital emergency departments and clinical ophthalmological services was therefore unaffected, which contrasts with the rest of the world [17, 38–41]. Our results demonstrated that there was no change in total ophthalmic presentation numbers to an outer metropolitan ED (Campbelltown Hospital) and an increase in ophthalmic cases presenting to a rural ED (Bowral and District Hospital) during the pandemic from March 1st to May 31st of 2020, in comparison to the same period in 2019. Surprisingly, both emergency departments saw a decrease in total general emergency presentations during this period in 2020. This latter finding mirrored results published in other studies of a similar observation period in Australia and New Zealand [5, 10, 11]; in which New Zealand had similar public health restrictions to NSW during March to May of 2020 [10]. With regards to ophthalmic presentations, there was an increase in non-urgent (Triage Category 5) presentations to both EDs, and a decrease in patients presenting with more serious issues such as visual disturbance. In Campbelltown Hospital, we found that there were fewer patients with ophthalmic conditions that did not wait to be seen, as well as a decrease in the length of stay for ophthalmic presentations, however these findings were not replicated in Bowral and District Hospital. On the other hand, Bowral ED saw a significant decrease in urgent (Triage Category 3) ophthalmic-related presentations.

Our finding that total ophthalmic presentation numbers remained unchanged for Campbelltown ED and increased at Bowral ED during COVID-19 is interesting, as it contrasts with other studies that had demonstrated a decline in ophthalmic ED presentations during the pandemic in Australia and other countries [13–19]. In particular, a similar analysis conducted in NSW by Kam et al. [15] demonstrated a fall of 16% in ophthalmic presentations during a similar time period of 29th March to 31st May at the metropolitan EDs of Western Sydney Local Health District (WSLHD), with return of presentation numbers to those of 2019 in June to July after easing of restrictions. Compared with the hospitals explored in their study, Campbelltown Hospital receives a greater number of patient presentations per year and is more peripherally located in Sydney [42], whilst Bowral and District Hospital is a rural hospital southwest of Sydney with fewer per annum presentations. A potential reason for the difference in ophthalmic presentation numbers in our study may be due to the difference in geographical location of these hospitals. There may have been differing perception of sites more affected by COVID-19 amongst the populace, secondary to proximity of identified cases to specific hospitals. This may have affected presentation patterns to these locations. Population relocation to more regional areas [43] due to increasing ability of citizens to work from home may have also resulted in an increase in ophthalmic presentations in outer metropolitan and rural hospitals.

Interestingly we found a significant increase in non-urgent (Triage Category 5) presentations to Campbelltown and Bowral EDs in 2020. A breakdown of discharge diagnoses for Category 5 presentations demonstrated that much of this increase was due to ocular surface trauma and eye pain (An additional table shows this in more detail [see Additional file 1 – Supplementary Table 1]). A reason for this may be the reduced access to primary care providers such as general practitioners (GP) or optometrists during the start of the pandemic in NSW and the limitations of GP telemedicine for these eye conditions [11]. The subjective patient-perceived urgency of their condition, due to ocular surface discomfort and pain, may therefore have caused an increase in ocular surface issues presenting to the emergency department. There is also evidence that since the start of the pandemic, ocular surface symptoms and dry eye have been exacerbated by displaced or poorly fitted face masks (Mask-Associated Dry Eye) and increased electronic display screen time [44–48]. An increase in home improvement projects due to stay-at-home orders may also correlate with the increase in ocular surface foreign bodies noted during the pandemic [18, 49, 50].

In Bowral, there was reduced access to the private ophthalmology referral practice for non-urgent cases during early March 2020 due to physician leave, whilst available ophthalmologists saw urgent cases only. This may also explain the increase in non-urgent ophthalmic presentations during the pandemic at Bowral ED. There was also an agreement between Bowral Hospital and the private ophthalmology practice for patients to be directly reviewed in the private ophthalmology rooms as part of the initial pandemic response in April 2020. Despite the introduction of this referral pathway, our data analysis demonstrated that there was no month-over-month change in ophthalmic presentation and referral patterns in 2020, and many patients presenting to Bowral ED with ophthalmic issues during COVID-19 were still being reviewed by emergency doctors, with only three patients lacking medical assessment documentation during our study period.

Consistent with previous studies, which have shown a drop in presentations for retinal detachments and other causes of visual acuity change or loss [13–15, 20, 51], there was a significant decrease in patients presenting with visual disturbances at Campbelltown Hospital ED in our study. There was also a decrease in visual disturbance presentations at Bowral Hospital, but this did not reach statistical significance. As aforementioned, ocular pain may have eclipsed visual disturbance as an important reason for patients to present to ED during COVID-19, despite fears of COVID-19 transmission [37]. A reduction in identification of patients with visual disturbance may have profound effects on the ongoing burden of vision loss as the pandemic resolves [52, 53].

There were significantly fewer patients in 2020 at Campbelltown Hospital who left the department before medical assessment, which was also reported by Kam et al. [15] This may have been due to a general decrease in case numbers across the emergency department, leading to fewer patients waiting, and was further supported in our study by the significant reduction in length of stay of patients presenting with ophthalmic issues at Campbelltown Hospital. On the other hand, the decrease in the length of stay and number of patients presenting with ophthalmic issues who did not wait for assessment at Bowral ED did not reach statistical significance. This is in the context of increased total ophthalmic-related presentations to this rural ED during 2020, and likely reflects its more streamlined ophthalmological referral pathway that existed prior to the pandemic, for which the private ophthalmology rooms are situated close to the hospital. In contrast to this, patients presenting to the more metropolitan Campbelltown Hospital must utilise private transportation to attend private ophthalmology rooms for review. There was no change in the number of patients being referred for ophthalmology follow-up at both hospitals, suggesting that referral patterns at both EDs did not change significantly with COVID-19, despite changes in types of presentations.

There are several limitations to this study. As a retrospective study of only ED coding data and documentation, the discharge diagnoses of each presentation may not be accurate and does not reflect the final diagnoses made by an ophthalmologist for those patients that received formal specialist review after discharge. However, our study replicated methods used by similar studies [15], which did not analyse documentation from ophthalmology follow-up visits. Similarly, by not assessing the characteristics of follow-up visits and the ongoing management of ophthalmic presentations in 2020, we were unable to determine whether COVID-19 restrictions had affected subsequent treatment times for acute ophthalmic conditions outside of the emergency department. There may also be potential unintentional changes in triage criteria in response to the COVID-19 pandemic, as suggested by the differences in discharge diagnoses per triage category in both EDs (An additional table shows this in more detail [see Additional file 1]). Finally, because the stay-at-home orders applied across NSW between March to May 2020 [24], this study did not assess how different levels of lockdown restrictions affected ophthalmic emergency presentations and management. The assessment of the effect of restrictions on ophthalmic ED cases during this period of the peak of the first wave of COVID-19 in NSW during 2020 can further be improved by a comparison with the number and type presentations in the months following easing of restrictions, in future studies.

Despite these limitations, our audit incorporates data from two EDs that differ by locality and acuity, which allowed for a comparison of the effect of COVID-19 on ophthalmic ED presentations across NSW. The inclusion of two hospital EDs also increased the sample size of our audit. We had also ensured that the coded discharge diagnoses were correct via individual patient chart reviews of their emergency department presentation, increasing the accuracy of our analysed data.

## Conclusions

Our findings suggest that the COVID-19 pandemic affected ophthalmic presentation trends heterogeneously in different parts of NSW despite the introduction of the same stay-at-home orders across the state [24]. In contrast to other studies, ophthalmic presentation numbers to the outer metropolitan Campbelltown Hospital ED remained unchanged across 2019 and 2020, whilst numbers increased at the rural Bowral and District Hospital ED. Both EDs had an increase in non-urgent presentations, but a decrease in more urgent issues such as visual disturbance. The overall need and access for acute ophthalmic services remained high at the geographical areas explored in this study, especially for non-urgent presentations, in contrast with other global locations [13–19, 38–41]. However, patients may have received inconsistent healthcare due to non-uniform changes in primary and tertiary healthcare service availability in different areas of NSW, such as reduced access to GPs, despite unrestricted access to emergency departments during the pandemic. Especially concerning is the reduction in patients presenting with visual disturbance to the emergency department, which may have significant long-term public health implications by increasing the burden of vision loss as we recover from the pandemic [53].

Compared with other countries, Australia has had fewer cases of COVID-19 per capita during the first wave of the pandemic [54], and the COVID-19 restrictions were comparatively weaker to other countries during this period [55]. It is therefore difficult to directly compare our results with studies conducted in other countries. Nevertheless, our study indicates that the pandemic has noticeably influenced patients seeking healthcare for ophthalmic conditions in Australia, as seen by the changes in urgent and non-urgent presentations during COVID-19. The varied effects of the pandemic on ophthalmic emergency presentations across NSW suggests that current methods of ophthalmic care delivery are inadequate during a pandemic. There may be a need for an alternative and more flexible ophthalmic healthcare delivery strategy linked to different geographical locations. With the rise in non-urgent (Triage Category 5) ophthalmic presentations in more regional communities, and a reduction in patients seeking care for more sight-threatening symptoms, alternative avenues of care such as tele-ophthalmology may become more ubiquitous and advantageous during and after the pandemic to improve management of these presentations. Our research group is currently developing such a tele-ophthalmology response for ED ophthalmic patients, with consideration of constraints presented by the COVID-19 pandemic.

## Supplementary Information

**Additional file 1: Supplementary Table 1.** List of discharge diagnoses organised by triage category for patients presenting to Campbelltown Hospital and Bowral and District Hospital Emergency Departments from March 1st to May 31st in 2019 and 2020. Tabulated data of the variety of discharge diagnoses for patients presenting to Campbelltown Hospital and Bowral and District Hospital Emergency Departments from March 1st to May 31st during COVID-19 and in the year prior to this (2019), organised by triage categories.

## Data Availability

All data generated or analysed during this study are included in this published article (and its supplementary information files). Further information is available from the corresponding author on reasonable request.

## Abbreviations

ED: Emergency Department
GP: General Practitioner
ICD-10: International Classification of Diseases 10th Revision
LGA: Local Government Area
NSW: New South Wales
PPE: Personal Protective Equipment
SWSLHD: South Western Sydney Local Health District
WSLHD: Western Sydney Local Health District
