# Supplementary material for: Impact of COVID-19 pandemic on ophthalmic presentations to an Australian outer metropolitan and rural emergency department: a retrospective comparative study
Source: BMC Ophthalmol. 2022 Jan 28;22:40. doi: 10.1186/s12886-022-02271-8 (PMC8796873; doi:10.1186/s12886-022-02271-8)
Supplement: Supplementary file 1 — Additional file 1: Supplementary Table 1. List of discharge diagnoses organised by triage category for patients presenting to Campbelltown Hospital and Bowral and District Hospital Emergency Departments from March 1st to May 31st in 2019 and 2020. Tabulated data of the variety of discharge diagnoses for patients presenting to Campbelltown Hospital and Bowral and District Hospital Emergency Departments from March 1st to May 31st during COVID-19 and in the year prior to this (2019), organised by triage categories. [file 12886_2022_2271_MOESM1_ESM.docx]

Impact of COVID-19 Pandemic on Ophthalmic Presentations to an Australian Outer Metropolitan and Rural Emergency Department: A Retrospective Comparative Study

King Fai Calvin Leung MD MMed^1^, Mojtaba Golzan PhD^2^, Chaminda Egodage FACEM^1^, Simon Rodda FACEM^3^, Richard Cracknell FACEM^1^, Peter Macken FRANZCO^4^, Shweta Kaushik FRANZCO PhD^2,5^

*^1^Department of Emergency Medicine, Campbelltown Hospital, Campbelltown, New South Wales, Australia*

*^2^Vision Science group, Graduate School of Health (GSH), University of Technology Sydney (UTS), Chippendale, New South Wales, Australia*

*^3^Department of Emergency Medicine, Bowral and District Hospital, Bowral, New South Wales, Australia*

*^4^Department of Ophthalmology, Bowral and District Hospital, Bowral, New South Wales, Australia*

*^5^Department of Ophthalmology, Campbelltown Hospital, Campbelltown, New South Wales, Australia*

**Corresponding author**: Dr. King Fai Calvin Leung

**Postal Address**: Department of Emergency Medicine

Campbelltown Hospital

Therry Road, Campbelltown, NSW 2560

**Email Address**: kfcalvinl@gmail.com

**Telephone Number**: +61 406 321 619

Supplementary Table 1. List of discharge diagnoses organised by triage category for patients presenting to Campbelltown Hospital and Bowral and District Hospital Emergency Departments from March 1^st^ to May 31^st^ in 2019 and 2020.

| Campbelltown Hospital | | | | | | Bowral and District Hospital | | | | | |
| --- | --- | --- | --- | --- | --- | --- | --- | --- | --- | --- | --- |
|  | **2019** | | | **2020** | |  | **2019** | | | **2020** | |
| Triage Category | **Diagnosis** | **n** | **Diagnosis** | | **n** | **Triage Category** | **Diagnosis** | **n** | **Diagnosis** | | **n** |
| 2 | Cerebrovascular accident  Chemical injury to cornea  Corneal abrasion  Periorbital cellulitis  Visual disturbance | 1  3  1  1  1 | Cerebrovascular accident  Chemical injury to cornea  Corneal abrasion  Drooping eyelid  Homonymous hemianopia  Retinal artery occlusion | | 1  2  1  1  1  1 | **2** | Low vision, one eye  Subconjunctival haemorrhage | 1  1 | Acute conjunctivitis  Hypertension  Multiple trauma | | 1  1  1 |
| 3 | Acute conjunctivitis  Allergic dermatitis of eyelid  Facial pain  Blunt injury of eye  Cerebrovascular accident  Chemical burn of eyelid AND/OR periocular area  Chemical injury to cornea  Conjunctival oedema  Corneal abrasion  Corneal ulcer  Did not wait for treatment  Discharge from eye  Disorder of eye  Eye pain  Eye swelling  Eyeball injury  Facial burn  Foreign body  HSV dendritic keratitis  Laceration of eyelid  Periorbital cellulitis  Stye  Superficial injury of eye region  Uveitis  Visual disturbance  Vitreous haemorrhage  Welder's flash | 3  1  1  2  1  1  3  1  3  1  4  3  1  3  1  1  1  3  1  1  3  1  1  1  10  1  1 | Acute conjunctivitis  Allergic reaction  Benign intracranial hypertension  Blunt injury of eye  Burn of eye  Chemical injury to cornea  Corneal abrasion  Corneal ulcer  Did not wait for treatment  Diplopia  Discharge from eye  Disorder of eyelid  Eye disorder  Eye mass  Eye pain  Foreign body  Laceration of eye  Laceration of eyelid  Low vision, one eye  Palpitations  Periorbital cellulitis  Retinal artery occlusion  Visual disturbance  Welder's flash | | 5  1  2  3  1  7  7  2  2  1  1  1  1  1  1  3  2  1  1  1  3  1  1  1 | **3** | Abscess of eyelid  Acute conjunctivitis  Acute orbital inflammation  Allergic conjunctivitis  Blepharitis  Blunt injury of eye  Cerebrovascular accident  Chemical injury to cornea  Corneal abrasion  Did not wait for treatment  Discharging wound  Disorder of eye  Eye pain  Eye symptom  Flu-like illness  Foreign body  Headache  Injury of eye region  Periorbital cellulitis  Temporal giant cell arteritis  Visual disturbance  Welder's flash | 1  2  1  1  1  1  1  1  4  2  1  1  1  1  1  3  1  1  3  1  2  1 | Allergic conjunctivitis  Chemical injury to cornea  Did not wait for treatment  Eye disorder  Eye pain  Eye swelling  Foreign body  Injury of sclera  Laceration of eye region  Laceration of eyelid  Panic attack  Vitreous haemorrhage | | 1  1  1  1  1  1  3  1  1  1  1  1 |
| 4 | Acute conjunctivitis  Allergic conjunctivitis  Allergic dermatitis of eyelid  Blepharitis  Blunt injury of eye  Burn of eye  Chemical burn of eyelid AND/OR periocular area  Chemical injury to cornea  Corneal abrasion  Corneal ulcer  Did not wait for treatment  Diplopia  Discharge from eye  Eye pain  Eye swelling  Eyeball injury  Foreign body  Headache  Infected chalazion  Laceration of eyelid  Left against medical advice  Orbital cellulitis  Periorbital cellulitis  Postoperative complication  Red eye  Stye  Subconjunctival haemorrhage  Victim of physical assault  Viral illness  Visual disturbance | 8  1  1  2  4  1  1  3  12  1  22  1  1  9  3  1  28  1  1  4  2  1  5  1  4  4  1  1  1  3 | Abscess  Acute conjunctivitis  Allergic conjunctivitis  Allergic reaction  Blunt injury of eye  Burn of eye  Chalazion  Chemical injury to cornea  Contact lens corneal oedema  Corneal abrasion  Corneal ulcer  Did not wait for treatment  Disorder of eye  Eye pain  Eye swelling  Facial injury  Foreign body  Headache  Herpes zoster ophthalmicus  Hyperglycaemia  Laceration of eyelid  Orbital cellulitis  Facial pain  Periorbital cellulitis  Red eye  Scleritis  Sixth cranial nerve injury  Stye  Subconjunctival haemorrhage  Superficial injury of eye region  Visual disturbance  Welder's flash | | 1  10  3  2  1  1  1  2  1  8  2  6  1  3  1  1  34  1  1  1  3  1  1  10  6  1  1  1  1  1  3  3 | **4** | Acute conjunctivitis  Blepharitis  Blunt injury of eye  Chemical injury to cornea  Closed fracture of orbital floor (blow-out)  Conjunctival injury  Corneal abrasion  Dacryocystitis  Did not wait for treatment  Eye pain  Foreign body  Headache  Keratitis  Laceration of eye region  Periorbital cellulitis  Red eye  Subconjunctival haemorrhage  Temporal giant cell arteritis  Tick bite  Visual disturbance  Welder's flash | 4  1  2  2  1  2  6  1  8  3  20  1  2  1  1  3  1  1  1  1  1 | Acute conjunctivitis  Angioedema  Blepharitis  Blunt injury of eye  Chemical injury to cornea  Corneal abrasion  Corneal ulcer  Did not wait for treatment  Eye pain  Eye swelling  Eye symptom  Foreign body  Furuncle of eyelid  Headache  Injury of sclera  Laceration of eye region  Laceration of eyelid  Periorbital cellulitis  Photokeratitis  Pterygium  Red eye  Stye  Subconjunctival haemorrhage  Visual disturbance | | 2  1  1  2  6  10  2  7  3  2  2  20  1  1  1  1  1  2  1  1  1  1  3  1 |
| 5 | Acute conjunctivitis  Allergic conjunctivitis  Blepharitis  Blunt injury of eye  Corneal abrasion  Corneal ulcer  Did not wait for treatment  Discharge from eye  Dry eyes  Foreign body  Headache  Laceration of eyelid  Stye  Viral illness | 2  1  1  2  2  1  12  1  1  8  1  4  2  1 | Acute conjunctivitis  Allergic reaction  Burn of eye  Chemical injury to cornea  Corneal abrasion  Corneal ulcer  Did not wait for treatment  Eye pain  Eye symptom  Foreign body  Hyphaema  Periorbital cellulitis  Red eye  Retinal tear  Scleritis  Stye  Superficial injury of eye region  Uveitis | | 4  1  1  1  4  1  9  8  1  21  1  2  2  1  1  1  1  1 | **5** | Did not wait for treatment  Eye pain  Foreign body | 1  1  1 | Chemical injury to cornea  Corneal abrasion  Did not wait for treatment  Disorder of lacrimal system  Foreign body  Periorbital cellulitis  Red eye | | 1  4  3  1  6  1  3 |
